# Supplementary material for: Influence of 2-Nitroimidazoles in the Response of FaDu Cells to Ionizing Radiation and Hypoxia/Reoxygenation Stress
Source: Antioxidants (Basel). 2023 Feb 6;12(2):389. doi: 10.3390/antiox12020389 (PMC9951954; doi:10.3390/antiox12020389)
Supplement: Supplementary file 1 [file antioxidants-12-00389-s001.zip › antioxidants-2159311-supplementary.pdf]

Supplementary information

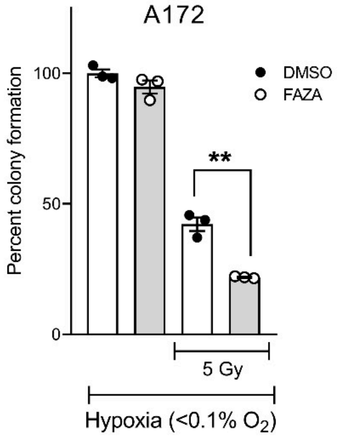

Figure S1. FAZA radiosensitizes hypoxic glioblastoma cells. A glioblastoma cell line (A172; kindly provided by Dr. Roseline Godbout, University of Alberta, Canada) was used to assess the hypoxic radiosensitization potential of FAZA. Hypoxic cells treated with FAZA (100 µM) for 4 h were subjected to 5 Gy of IR, and allowed to recover and form colonies for 14 days. Colonies were stained with crystal violet, counted and normalized to the colony counts in the unirradiated DMSO treated conditions. A shorter duration was chosen to minimize the cytotoxic effects of the drug to better illustrate its effect on the radiation arm.

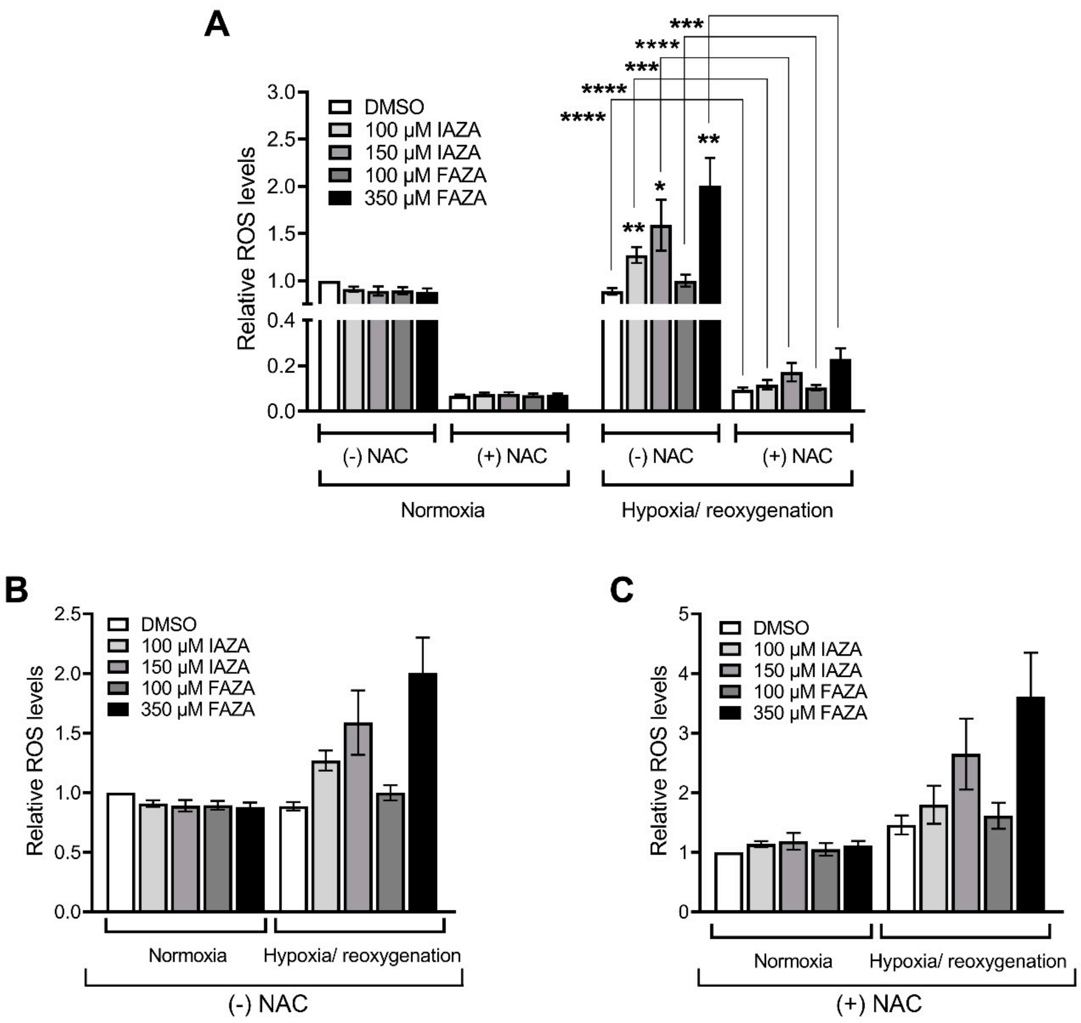

Figure S2. High levels of ROS in IAZA-/FAZA-treated hypoxic/reoxygenated cells. (**A**) A significant increase in H_2O_2 levels was found in drug-treated reoxygenated cells, which could be quenched if reoxygenation was carried out in the presence of 3 mM N-acetylcysteine (NAC). Relative fold change in cellular ROS levels in cells not subjected to NAC **(B)** and 3 mM NAC **(C)** retained a similar trend. Data represent the mean± S.E.M. from three independent replicates.
